# Decoding Non-Invasive Electroencephalography Signal via a Two-Discriminator Adversarial Network

**DOI:** 10.3390/s26031074

**Published:** 2026-02-06

**Authors:** Xuguang Liu, Changyi Yu, Ye Li, Xin Zhang, Xiu Zhang

**Affiliations:** 1 Tianjin Key Laboratory of Wireless Mobile Communications and Power Transmission, Tianjin Normal University, Tianjin 300387, China; liuxuguang1215@163.com (X.L.);; 2 College of Artificial Intelligence, Tianjin Normal University, Tianjin 300387, China

**Keywords:** electroencephalography, non-invasive, emotion recognition, deep learning, domain adversarial neural network

## Abstract

Electroencephalography (EEG), as a typical non-invasive biosensing signal, reflects individual emotional changes by recording the brain’s neural activity in response to various external stimuli. However, the significant differences in brain activity among individuals and the complex interrelationships between EEG channels notably hinder the accuracy of emotion decoding in non-invasive biosensing scenarios. To address this challenge, this paper proposes a two-discriminator domain adversarial neural network method (TD-DANN). The proposed method aims to obtain more generalized and individualized emotion feature representations through adversarial learning. Specifically, graph convolution is utilized to extract features from EEG signals. By modeling the EEG channels as graph nodes, the adjacency matrix can be dynamically learned to capture the complex relationships between different channels during emotion generation. Moreover, we design a domain discriminator and an individual discriminator. The domain discriminator is used to minimize the difference in feature distribution between the source and target domains. It is able to obtain discriminative features with universality. The individual discriminator is used to learn discriminative features consistent with the individual’s brain activity. It can enhance the adaptability to the individual’s emotion. The experimental results show that the TD-DANN achieves promising recognition accuracies of (98.45 ± 2.38)% and (89.45 ± 5.87)% for subject-dependent and subject-independent experiments on the SEED dataset, respectively. The proposed method attains recognition accuracies of (84.40 ± 8.70)% and (77.13 ± 7.97)% for subject-dependent and subject-independent experiments on the SEED-IV dataset, respectively. These results validate the effectiveness of the TD-DANN in the emotion decoding problem.

## 1. Introduction

Emotion recognition (ER) is a fundamental research topic in affective computing and plays an important role in human–computer interaction and intelligent systems. From the perspective of signal sources, emotion recognition methods can be broadly categorized into approaches based on external behavioral signals and internal physiological signals. External behavioral signals, such as facial expressions, speech, and body gestures, are intuitive but can be intentionally disguised, which limits their reliability in practical applications [1,2]. In contrast, internal physiological signals reflect intrinsic biological responses and are more difficult to conceal [3]. These signals could be acquired using invasive or non-invasive techniques. Among them, electroencephalography (EEG) and magnetoencephalography (MEG) are non-invasive techniques for acquiring neural activity signals and have been widely used in affective computing research [4,5]. Due to the advantages of high temporal resolution, relatively low cost, and the ability to directly measure cortical activity, as well as the high correlation between EEG signals and human emotional activity, EEG has become one of the most commonly utilized modalities for ER [6].

When processing EEG signals in emotion recognition tasks, EEG features can be categorized into two types: time-domain features and frequency-domain features [7]. Time-domain features are generally concerned with the dynamic pattern of EEG signals over time. Typical time-domain features are fractal dimension features [8], Hjorth features, and higher-order crossover features [9]. Frequency-domain features analyze the energy distribution of EEG signals from the frequency perspective; the most common method is filtering the EEG signals into different frequencies. Then we could extract information in the specific frequency bands for emotion recognition. Researchers have proposed various methods for emotion classification. Wang et al. used support vector machines (SVM) for emotion classification [10], and achieved an accuracy of (83.99 ± 9.72)% in subject-dependent experiments on the SEED dataset. Zheng et al. used deep belief networks (DBN) for emotion classification [11], while the recognition accuracy in a subject-dependent experiment on the SEED dataset reached (86.08 ± 8.34)%. They also investigated the key channels and frequency bands in emotion recognition using differential entropy (DE) [12] features and power spectral density (PSD) [13] features. However, these methods ignore the complex relationship between different EEG channels and the differences in EEG features of different individuals, which can also greatly affect the recognition accuracy, especially in subject-independent experiments.

Therefore, in order to fully consider the influence of the interrelationships between different EEG channels on emotion classification, researchers have tried to explore the spatial relationship between EEG channels. Wang et al. proposed an ER method based on the spatial relationship of EEG channels [14]; they mapped the EEG channel positions onto a two-dimensional plane and combined the time-frequency information to construct a four-dimensional convolutional neural network (4DCRNN) for emotion classification. This method effectively captures the interdependence between different EEG channels by jointly modeling the spatial location and time-frequency features of the EEG channels and improves the ER accuracy. Yu et al. employed a capsule network integrated with an attention mechanism to extract multi-scale spatial features, thereby mapping EEG signals into a high-dimensional space [15]. A dynamic graph convolutional neural network (DGCNN) is proposed in [16], which obtains a more in-depth relationship between EEG channels by considering EEG channels as nodes in a graph and dynamically updating the neighboring relationships between nodes during training, further improving the ER accuracy.

To address the feature differences among different individuals, researchers have focused on domain adaptation. Lan et al. compared several common domain adaptation methods [17]. The methods are maximum independent domain adaptation, transfer component analysis, and subspace alignment. Their study shows that domain adaptation can effectively improve the ER accuracy, especially when the source and target domains come from different individuals with large differences in feature distributions. Li et al. proposed a bi-hemispheric domain-adversarial model [18]. Their method divided the EEG channel into two hemispheres and performed domain-adversarial training on each hemisphere separately, and then performed global domain-adversarial training. This strategy effectively reduces the distribution gap between source and target domain features. The strategy constructs a global feature representation with stronger generalization ability, thus improving the accuracy of emotion recognition.

However, solely relying on domain adversarial training, although it can effectively reduce the distributional differences between source and target domain features, tends to ignore the variability among different individuals. Each individual has a unique pattern of EEG signals. The unique emotional features play an important role in ER tasks. How to preserve each individual’s unique emotional pattern in the cross-individual emotion recognition becomes a key factor. To address this problem, we designed an individual discriminator to introduce individual discrimination to distinguish EEG features from different individuals during model training. It can capture individual-specific emotion feature representations and enhance the model’s adaptability to individual differences. In addition, in order to better mine the interrelationships among EEG channels, we also adopt graph convolution operation as a feature extractor. By learning the complex topological structure among EEG channels, we can effectively extract deep features that are closely related to emotion states. This potentially improves the overall performance of the ER task.

In summary, the contributions of the paper are listed as follows:

(1) Proposing a two-discriminator domain adversarial neural network (TD-DANN) method for emotion recognition: The method uses graph convolution as a feature extractor to effectively capture the complex relationships among EEG channels. The domain discriminator is able to obtain generalized discriminative features through reducing the feature distribution gap between the source domain and target domain.
(2) Introducing an individual discriminator: The individual differences in cross-subject ER tasks are addressed by incorporating an individual discriminator into the model. By learning the unique EEG features of different individuals, the model enhances its adaptability to individual differences, thereby improving emotion recognition accuracy.
(3) Extensive experiments on public datasets: Comprehensive experiments are conducted on the publicly available SEED and SEED-IV datasets. The experiments include both subject-dependent and subject-independent paradigms. Through analyzing the results, it is found that individual-specific emotion features significantly affect the ER accuracy. The ER accuracy is effectively improved through the effective discrimination of these features.

The rest of the paper is organized as follows: a brief overview of the current state of research on graph convolution and domain adversarial networks is given in Section 2. The proposed method in this paper is described in detail in Section 3. Section 4 presents the experiments and results. Section 5 concludes the paper.

## 2. Background

In this section, we review related works on graph convolution and domain adversarial neural networks.

### 2.1. Graph Convolutional Network

Graph convolutional network (GCN) combines local graph structure and feature information to perform effective convolution operations on graph-structured data. The concept of GCN was first introduced by Kipf et al. to address the limitations of traditional neural networks when handling non-Euclidean data [19]. The GCN learns a richer representation of nodes by performing convolutional operations on the convolution operation on the neighborhood information of a node [20]. It is able to combine the topological information of the graph structure with the features of the nodes to learn a richer representation of the nodes, making each node not only rely on its own features but also be able to integrate the features of its neighboring nodes, thus improving the performance of the model [21]. In [22], the authors introduced a fast localized spectral filter to improve GCN. EEG signals are multidimensional data consisting of multiple EEG channels, and there are complex spatial relationships between these channels. Zhang et al. combined dynamic graph convolutions with attention mechanisms to evaluate the sensitivity of different EEG features to emotional changes, delving into their impact on emotion recognition and providing richer, more accurate feature information [23]. Song et al. successfully applied graph convolution to the EEG emotion recognition task by treating each EEG channel as a node of a graph [16]. This approach enhances the machine’s understanding of the complex relationships between EEG channels through graph convolution operations, which further improves the accuracy of emotion classification. Zhang et al. further improved this approach by proposing a Sparse Dynamic Graph Convolutional Neural Network (SparseDGCNN) model [24], which optimizes the representation of the graph by applying sparsity constraints to the graph, thus improving the emotion recognition performance.

### 2.2. Domain Adversarial Neural Network

Domain adversarial neural network (DANN) was first proposed in [25] to address the problem of differences in the data distributions between source and target domains; the core idea of DANN is to learn a shared feature distribution through adversarial training in the source and target domains, while ensuring that this shared feature can effectively classify the source domain. In EEG emotion recognition, individual differences in brainwave signals, even in response to the same stimuli, can affect the accuracy of emotion classification, especially in subject-independent experiments. Jin et al. introduced DANN into EEG emotion recognition [26] and improved the accuracy of emotion recognition, benefiting from the method’s good adaptability to the distribution differences between source and target domains. Inspired by neuroscience research that found asymmetric properties of the left and right brains in emotional responses, Li et al. proposed a bi-hemispheric domain adversarial neural network (BiDANN) model [18]. The model contains one global and two hemispheric domain discriminators, which are trained for domain adversarial training of features from the left and right brains, respectively, reducing the distribution difference between the source and target domains in each hemisphere. The results showed that BiDANN significantly improved emotion recognition accuracy compared to the original DANN. Du et al. proposed an emotion recognition model based on the attentional mechanism and DANN [27], called Attention-based LSTM with Domain Discriminator (ATDD-LSTM). This model assumes that not all EEG channels play equal roles in emotion recognition tasks, so LSTM was introduced to focus on EEG channels that are important for emotion classification by weighting the features of different channels, again based on the introduction of a domain discriminator, which improves the model’s classification ability.

## 3. Methodology

**Figure 1:**
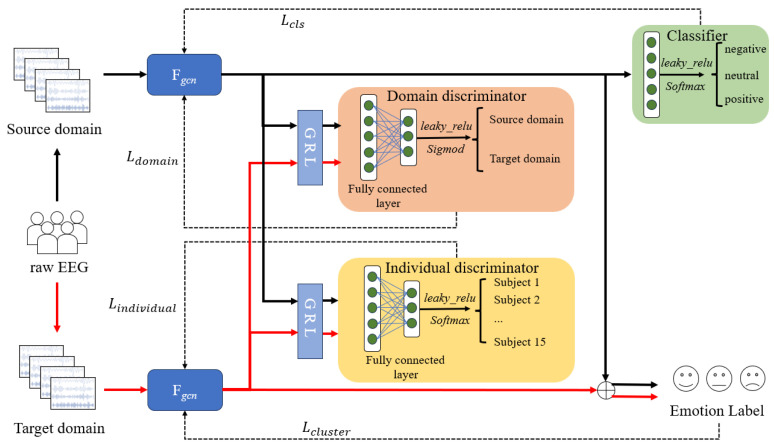
Structure of the proposed TD-DANN method. $F_{gcn}$ is the graph convolutional feature extractor, which is given in Figure 2. GRL is the gradient reverse layer. The red line represents the target domain data, and the black line represents the source domain data.

In this section, the TD-DANN method is introduced in detail. The overall framework of the TD-DANN method is shown in Figure 1. The method consists of a graph convolutional feature extractor $F_{gcn}$, a domain discriminator $D_{D}$, an individual discriminator $D_{i}$, two gradient reverse layers GRL, and a classifier C. Next, we will describe the model in detail from several aspects, such as the graph convolutional feature extractor, domain discriminator and individual discriminator.

### 3.1. Graph Convolutional Network Feature Extractor

**Figure 2:**
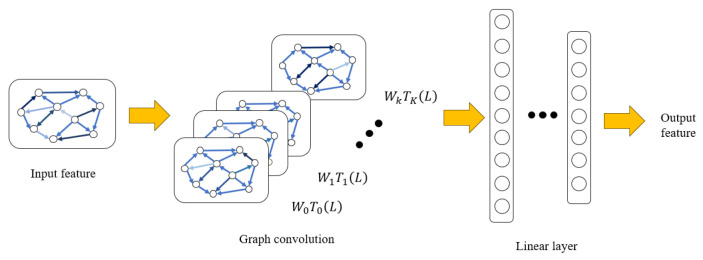
The feature extraction process of graph convolutional feature extractor $F_{gcn}$ in TD-DANN.

EEG signals exhibit complex spatial dependencies across different brain regions, and the relationships among EEG channels are inherently non-Euclidean. Traditional convolutional neural networks are limited in modeling such irregular spatial structures, as they rely on fixed grid-based representations. To address this limitation, we adopt a graph convolutional network to explicitly model inter-channel relationships and extract spatially informed EEG features so as to extract discriminative features and improve the ER accuracy. The extraction procedures are presented in Figure 2.

We represent the EEG signals as a graph G=(V,E), where *V* is the set of nodes, containing all EEG channels, and *E* is the set of edges between nodes in *V*. Referring to the conclusions drawn from previous studies [11,28], the corresponding node feature vector is then constructed using DE features extracted from multiple frequency bands to better capture frequency-specific neural activity associated with emotional states. Let $X\in R^{N\times F}$ denote the input feature matrix, where N=|V| is the number of EEG channels and F represents the feature dimension of each channel.

Unlike approaches that rely on predefined electrode layouts or static adjacency matrices, we construct an adaptive adjacency matrix that is jointly optimized with the network parameters. Specifically, the adjacency matrix $A\in R^{N\times N}$ is initialized based on learnable parameters and updated through backpropagation during training. This allows the graph structure to dynamically capture inter-channel dependencies that are most relevant to emotion recognition, where each element $A_{ij}$ represents the strength of the connectivity between channel *i* and channel *j*. Initially, the adjacency matrix is randomly created by the Xavier initialization method to ensure stable training of the network. The initialization method is as follows:

(1) $$A∼u\left(-\frac{1}{\sqrt{N}},\frac{1}{\sqrt{N}}\right)$$

By learning the adjacency matrix in a data-driven manner during the model training phase, the proposed GCN can adapt to the EEG patterns of a particular individual. Although the graph structure is shared across subjects during training, the learned inter-channel relationships reflect statistical regularities extracted from EEG responses of different individuals. Thus, the adaptive adjacency matrix enhances the cross-subject generalization of the model while preserving the underlying spatial features. Since the matrix *A* is symmetric, the optimization goal of the model during training is to learn a symmetric matrix to ensure the integrity of the graph structure. To ensure the stability and validity of the graph convolution operation, before using the adjacency matrix *A*, we perform symmetric normalization on it so that the features of each channel can be uniformly propagated in the graph structure, which avoids the overconcentration of information in some specific channels. The specific symmetric normalization method is as follows:

(2) A′=ReLU(A)

(3) $$L=D^{-\frac{1}{2}}A^{\prime }D^{-\frac{1}{2}}$$

where $A^{\prime }$ is the adjacency matrix attained by applying the ReLU function to *A*. This makes the matrix elements nonnegative. The degree matrix *D* is defined as $D_{ii}=\Sigma_{j}A_{ij}^{\prime }$, representing the degree of *i*. The normalized adjacency matrix *L* takes the form of the graph Laplace matrix, which can effectively reduce the imbalance problem during information propagation and ensure that the information can be uniformly propagated in the graph.

In order to improve the expressive power of graph convolution, we adopt the Chebyshev polynomial approximation method for graph convolution operation, and the recursive formula of the Chebyshev polynomial is given by

$$T_{0}\left(L\right)=I,T_{1}\left(L\right)=L$$

(4) $$T_{k}\left(L\right)=2LT_{k-1}\left(L\right)-T_{k-2}\left(L\right),k\geq 2$$

where *I* denotes the identity matrix, *L* denotes the normalized Laplacian matrix, and $T_{k}\left(L\right)$ represents the k-th order Chebyshev polynomial. With the Chebyshev polynomials, we are able to perform efficient filtering and feature extraction on the graph signal. The formula for each layer of graph convolution operation is as follows:

(5) $$Z=\sum_{k=0}^{K-1}T_{k}\left(L\right)XW_{k}$$

where K is the Chebyshev polynomial order, $W_{k}\in R^{F\times F^{\prime }}$ denotes the weight matrix of the k-th order polynomial, and *Z* denotes the output feature matrix. After the graph convolution operation, we nonlinearly transform the results with the Leaky ReLU activation function. This function is able to improve the expressiveness of the model.

### 3.2. Domain Discriminator and Individual Discriminator

To enhance the model’s generalization capability and adaptability to individual differences among different individuals, this paper introduces a domain discriminator and an individual discriminator to optimize the feature representation through adversarial training. The domain discriminator and individual discriminator impose constraints on the feature extractor from the dimensions of domain alignment and individual differences, respectively. This further improves the model’s generalization capability and adaptability to individual differences for ER tasks.

The primary task of the domain discriminator is to discriminate the distribution of features in the source domain and target domain. It can instruct the feature extractor to narrow the distribution gap between these two in the backpropagation. Denote the source domain feature as $f_{s}$. Denote the target domain feature as $f_{t}$. Specifically, the $f_{s}$ and the $f_{t}$ processed by the extractor will be concatenated as $f=\left\{f_{s},f_{t}\right\}$, and then input to the domain discriminator via GRL. The domain discriminator would output a binary probability. The probability indicates that the input feature belongs to the source domain or the target domain. For a source domain feature $f_{s}$, the domain discriminator is expected to have an output close to 1, indicating that it is correctly categorized. For a feature of the target domain, the output is expected to be close to 0, indicating that it is correctly categorized. For a feature of the target domain, the output is expected to be close to 0, indicating that it is correctly categorized as the target domain. We compute the loss for domain adversarial using a binary cross-entropy (BCE) loss function,

(6) $$L_{\mathrm{domain}\;}=\frac{1}{2}\left(BCE\left(d_{S},1\right)+BCE\left(d_{t},0\right)\right)$$

where $d_{S}$ and $d_{t}$ are the output probabilities of the discriminator for features belonging to the source domain and the target domain, respectively. And 1 and 0 correspond to the true domain labels of the features. The BCE is computed as

(7) $$\mathrm{BCE}\left(y,\hat{y}\right)=-\frac{1}{N}\sum_{i=1}^{N}\left[y_{i}\log\left({\hat{y}}_{i}\right)+\left(1-y_{i}\right)\log\left(1-{\hat{y}}_{i}\right)\right]$$

where $y_{i}$ is the true label of sample $i,{\hat{y}}_{i}$ is the predicted probability, and *N* is the number of training samples.

By minimizing $L_{\mathrm{domain}}$, the domain discriminator prompts the feature extractor to extract more domain-independent features in the process of distinguishing the source domain and the target domain. In addition, the GRL transfers the negative gradient of the domain adversarial loss back to the feature extractor, thus optimizing its feature representation and making the extracted features as indistinguishable as possible between the source domain and the target domain, thereby narrowing the distribution gap between them.

Due to the inherently individual-specific characteristics of EEG signals, the objective of the individual discriminator is to capture inter-individual differences relevant to emotion recognition through adversarial learning. This design prevents the domain alignment process from excessively suppressing individual-specific emotional patterns, thereby enhancing the model’s ability to adapt to diverse brain activity characteristics across subjects. The input to the individual discriminator is also the spliced features $f=\left\{f_{s},f_{t}\right\}$ of the source domain and the target domain, which are then fed back to the individual discriminator via GRL. The individual discriminator would output a probability. The probability distribution of the samples belonging to different individuals, and we compute its individual adversarial loss using the cross-entropy loss function, which is

(8) $$L_{individual}=CE\left(p,id\right)$$

where *p* is the output probability distribution of the feature representation by the individual discriminator and id is the ground truth individual label of the sample. The cross-entropy loss CE is calculated as

(9) $$CE\left(y,\hat{y}\right)=-\sum_{i=1}^{C}y_{i}\log\left({\hat{y}}_{i}\right)$$

where $y_{i}$ denotes the ground truth label, ${\hat{y}}_{i}$ is the probability that the sample is predicted to be in the category *i*, and *C* is the number of classes in the dataset.

By minimizing $L_{\mathrm{individual}}$, the individual discriminator encourages the feature extractor to learn feature representations that can effectively distinguish between different individuals. Meanwhile, the gradient inversion layer performs adversarial optimization of the feature extractor so that the extracted features can both capture inter-individual differences and highly correlate with the emotional state, thus improving the ability of the model to recognize specific emotion features of an individual.

### 3.3. Domain Adversarial Learning

The feature extractor and discriminators are connected through a Gradient Reversal Layer (GRL). The role of GRL is to invert the gradient of the features during backpropagation, forcing the feature extractor to learn domain-insensitive features. Conventional GRLs maintain a fixed gradient inversion strength during training, which can lead to large biases in the features learned by the model in the early stages of training. We design the GRL with dynamically tuned parameters to dynamically adjust the gradient inversion strength. Specifically, this layer smoothly adjusts the change in gradient inversion strength using a Sigmoid function based on the current number of iterations. It reduces the inversion strength at the early stage of training. The proposed method can first learn the available features between the source domain and the target domain and gradually increase the inversion strength as the training progresses to motivate the feature extractor to learn the shared features that are domain-independent, thus facilitating the alignment between the source domain and the target domain. The gradient inversion strength coefficient coeff is calculated as follows:

(10) $$coeff=\frac{2(h_{i}-l_{o})}{1+\exp\left(-\alpha \cdot \frac{iter\_num}{max\_iters}\right)}-\left(h_{i}-l_{o}\right)+l_{o}$$

where $h_{i}$ and $l_{o}$ denote the upper and lower limits of the gradient reversal strength, respectively, α is a parameter controlling the rate of change in the reversal strength, iter_num denotes the current number of iterations, and max_iters denotes the maximum number of iterations. As the training progresses, the value of coeff gradually approaches hi, thus increasing the intensity of gradient inversion in the later stages of training and prompting the feature extractor to learn the feature representation shared across domains.

In the source domain, a supervised emotion classification method is used to optimize the emotion recognition ability of the method by minimizing the emotion classification loss. The specific emotion classification loss function is

(11) $$L_{cls}=BCE\left(y,\hat{y}\right)$$

where *y* is the ground truth label and $\hat{y}$ is the probability predicted by the method. For the source domain features, we want the model to accurately predict the emotion category, making the model’s output close to the true label.

In the target domain, an unsupervised method is used for emotion classification. Since the labels in the target domain are unavailable, we optimize the clustering effect of feature representations by selecting effective samples in the target domain. Specifically, the model chooses the most representative samples in the target domain by calculating the similarity matrix, and calculates the clustering loss from these samples. The goal is to enhance the clustering ability in the emotion classification task by minimizing the clustering loss, which is calculated as follows:

(12) $$L_{\mathrm{cluster}\;}=\frac{1}{N_{\mathrm{selected}\;}}\sum_{i=1}^{N_{\mathrm{selected}\;}}\left[BCE\left(d_{T}^{i},1\right)\right]$$

where $N_{\mathrm{selected}}$ denotes the number of selected target-domain samples, and $d_{T}^{i}$ represents the output of the target discriminator for the *i*-th selected sample. The target-domain clustering loss $L_{\mathrm{cluster}}$ is designed to encourage consistency and compactness among selected target samples in the discriminator output (decision) space rather than directly in the feature space. Specifically, target-domain samples with high predicted emotion confidence are first selected, resulting in $N_{\mathrm{selected}}$ samples. For each selected target sample, the corresponding discriminator output $d_{T}^{i}$ is encouraged to approach the same target state by minimizing the binary cross-entropy loss $\mathrm{BCE}(d_{T}^{i},1)$. This objective implicitly promotes clustering behavior by reducing the variance of discriminator outputs among selected target samples, thereby enhancing the compactness of target-domain representations in the decision space. The normalization term $N_{\mathrm{selected}}$ ensures stable optimization across different mini-batches. By enforcing consistency in the discriminator output space, the proposed clustering loss contributes to more robust target-domain adaptation and ultimately improves emotion recognition performance.

Combining all the above losses, the total loss function of the proposed method can be defined as follows:

(13) $$L_{\mathrm{total}\;}=L_{\mathrm{cls}\;}+L_{\mathrm{domain}\;}+L_{\mathrm{individual}\;}+\lambda L_{\mathrm{cluster}}$$

where λ is the weight used to adjust the clustering loss. By minimizing this total loss function, the model is able to align features in the source domain and the target domain, and effectively capture the inter-individual variability features, thus improving the generalization ability and personalized adaptation of the ER task.

## 4. Experimental Results and Analysis

In this section, we will conduct experiments on two publicly and widely used datasets in EEG emotion recognition tasks: SEED [11] and SEED-IV [28], to validate the performance of the proposed method.

### 4.1. Dataset and Experimental Setting

The SEED dataset is an EEG dataset developed by Shanghai Jiao Tong University that is widely used in emotion recognition research. The dataset contains EEG data from 15 healthy subjects (aged between 23 and 26 years old). In the experiment, subjects induced target emotions by watching 15 carefully selected video clips containing three emotion categories: positive, negative, and neutral. After watching each video clip, subjects were required to complete a self-assessment questionnaire to rate their emotional state, and these ratings were used as emotion labels.

The SEED-IV dataset is an extension of the SEED dataset to further explore the challenges of multi-class emotion recognition, which contains EEG data from 15 healthy subjects (aged between 18 and 24 years old). Subjects evoked target emotions by watching 24 video clips containing four emotion categories: happy, negative, fear, and neutral.

The DE features were extracted as the emotion features to validate the proposed EEG emotion recognition model. To fully evaluate the effectiveness of our proposed TD-DANN model, we designed two experimental scenarios, including subject-dependent and subject-independent experiments. In this model, we performed non-overlapping slices of the EEG signals provided by the dataset in a sliding window of 0.5 s, with each segment containing 310 features (i.e., 5 frequency bands × 62 channels). Therefore, the data size for the SEED dataset is 3 sessions × 15 subjects × 3394 samples × 310 features, and the data size for the SEED-IV dataset is 3 sessions × 15 subjects × 851/832/822 samples × 310 features. We used the RMSprop optimizer for network training, with the learning rate set to 0.0005, and L2 regularization with a weight of 0.0001 was used to prevent overfitting. The Chebyshev order in the graph convolutional feature extractor was set to K=3, the parameters $h_{i}$ and $l_{o}$ in Equation (8) were set to 0.9 and 0.5, respectively, the batch size was 96, and the epoch was 1000. The above parameters were consistent across subject-dependent and subject-independent experiments; all experiments were trained on NVIDIA GeForce RTX 4060 GPUs using the Pytorch API of CUDA 12.4.

### 4.2. Subject-Dependent Experiments

In the subject-dependent experiments, we validate the proposed model using the same experimental protocol as in [18,29,30,31] to comprehensively assess its performance and advantages. Specifically, for the experimental design in the SEED dataset, we divide the 15 EEG data from a single subject in a single session into two parts, with the first 9 experiments as the source domain and the remaining 6 experiments as the target domain. Similarly, in the SEED-IV dataset, the 24 EEG data contained in each session of each subject were divided into the first 16 experiments as the source domain and the remaining 8 experiments as the target domain. This division ensures the systematic and comparable nature of the experiments, and also fully validates the generalization ability of the model in cross-domain learning scenarios.

We trained the method on the entire session data of all subjects and computed the classification accuracy for each subject in the dataset. The final, average classification accuracy and standard deviation of all subjects are taken as the metrics to evaluate the model performance. In addition, the average training time for individual subjects in subject-dependent experiments for the SEED and SEED-IV datasets was about 1678 s and 426 s. Table 1 gives the comparison of the experimental results of our proposed TD-DANN model with several existing methods on the SEED and SEED-IV datasets. The existing methods include traditional machine learning methods such as SVM and DBN as well as deep learning methods such as DANNs, DGCNN, RGNN, BiDANN, BiHDM and PR-PL. The experimental results of Table 1 show that the TD-DANN model achieves better results in all cases. It attains 98.45±02.38% and 84.40±08.70% accuracy on the SEED (three-classification) and SEED-IV (four-classification) datasets, respectively, and exhibited lower standard deviations. This indicates that the proposed method has higher stability across subjects and can be effectively adapted to emotion classification tasks. This may be attributed to our proposed individual discriminator, which effectively captures the differential features among different individuals and improves the model’s adaptation to the characteristics of different individuals’ brain activities.

### 4.3. Subject-Independent Experiments

In subject-independent experiments, we use the same experimental protocol as in [18,29,30,31], i.e., the Leave-One-Subject-Out (LOSO) cross-validation strategy to evaluate the performance of our proposed TD-DANN model. Specifically, in each experiment, we use the full session data of one subject as the target domain and the full session data of the other 14 subjects as the source domain.

We took turns using each subject as the target domain and calculated the average classification accuracy and standard deviation as performance evaluation metrics for the model. Table 2 summarizes the experimental results. Table 2 shows the comparison of subject-dependent experimental accuracy (ACC) and standard deviation (STD) of models on SEED and SEED-IV datasets. In addition, the average training time for individual subjects in subject-independent experiments for the SEED and SEED-IV datasets for the LOSO experiments was about 2220s and 505s. In Table 2, the proposed TD-DANN method is compared with several existing methods on the SEED and SEED-IV datasets. The proposed method achieves better results on both datasets, with an accuracy on SEED of (89.45 ± 5.87)% and (77.13±7.97)% on the SEED-IV dataset. The experimental results indicate that the proposed method performs well in the cross-individual ER task by complementing the individual differences that cannot be effectively addressed by the traditional domain discriminator with the individual discriminator. The proposed method captures the shared features across domains while paying more attention to the diversity among individuals.

Comparing the results of Table 1 and Table 2, it is observed that the subject-dependent experimental results are significantly better than the subject-independent experimental results, with a difference ranging from 7% to 15%. This is not surprising; this phenomenon occurs because the EEG signal has a strong subjectivity. Different subjects will have different EEG activities under the same external stimuli, while the model in the subject-independent experiments has not learned the samples of the target domain in the training stage, and cannot effectively catch the emotion features in the target domain, so as to make an accurate classification. This phenomenon makes the subject-independent ER task of EEG signals more challenging, and the transfer learning method, including domain adversarial learning, is obviously more suitable for solving this kind of problem with different distributions of the source domain features and the target domain features, which is also verified by the experimental results. The subject-independent experimental results of DANN, BiHDM, BiDANN, and PR-PL models that use domain adversarial ideas significantly outperform those of SVM, DGCNN, and other models that do not include domain adversarial ideas.

Subject-independent emotion recognition is a critical requirement for practical EEG-based affective computing systems, as collecting subject-specific calibration data is often time-consuming and impractical in real-world applications. In such scenarios, models are required to generalize effectively to unseen subjects while maintaining stable recognition performance. The proposed TD-DANN explicitly addresses this challenge by jointly reducing inter-subject distribution discrepancies and preserving individual-specific EEG characteristics. The performance improvement observed in the subject-independent setting benefits from the introduction of the individual discriminator, which enables a better balance between general emotion-related EEG representations and individual-specific neural patterns. This design helps prevent situations in which the recognition accuracy of certain subjects is significantly lower than that of others. Experimental results on both the SEED and SEED-IV datasets demonstrate that TD-DANN consistently outperforms competing methods in subject-independent settings, highlighting its strong generalization capability across different subjects.

### 4.4. Ablation Experiment

**Figure 3:**
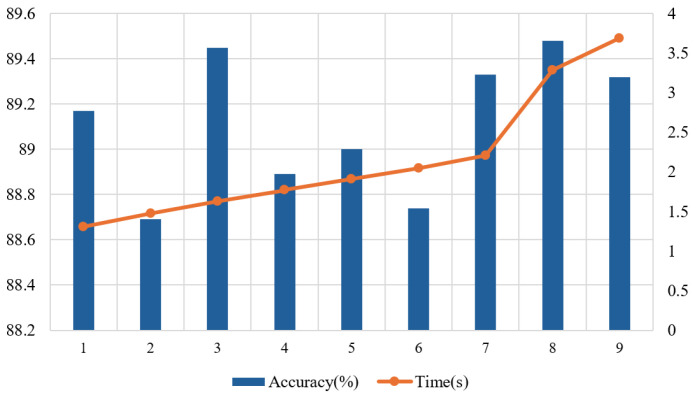
The accuracy (%) and training time (s) per epoch of the model with different numbers of layers K in the graph convolution feature extractor.

We explored the effect of the layer number K of the graph convolution feature extractor on the performance of the proposed method. Using the subject-independent experiment on the SEED dataset as an example, we tested the classification accuracy and the training time for a single epoch with different values of K. As shown in Figure 3, the model accuracy fluctuates between 88.69% (K = 2) and 89.48% (K = 8) when K is between 1 and 9, and the training time for a single epoch increases significantly as K increases. This is because a larger K increases the computational complexity of the graph convolutional layer, which significantly raises the time cost of the model. To balance the model performance and computational efficiency, we chose K=3 as the Chebyshev polynomial order of the graph convolutional feature extractor in the model. This choice ensures that the model’s performance is close to optimal while effectively reducing the time cost, making the model more efficient and practical, which is a reasonable compromise between performance and efficiency.

To assess the effect of different components in the proposed model, we conducted ablation experiments using the subject independent experiments on the SEED and SEED-IV datasets. Specifically, we compared the performance of the proposed model under the following ablation settings: (1) removing the individual discriminator while keeping the domain discriminator and the graph convolution feature extractor; (2) replacing the graph convolution operation with a multilayer perceptron consisting of two linear layers; (3) simultaneously performing (1) and (2). The results of the ablation experiments are given in Table 3.

After removing the individual discriminator, the accuracy results on the SEED and SEED-IV datasets significantly decrease. This indicates that the individual discriminator plays a crucial role in capturing differential inter-individual emotion features by effectively modeling the emotion representations of different individuals through the individual discriminator, which is important in the cross-individual ER task. It is found that after using the multilayer perceptron instead of the graph convolutional feature extractor, the model decreased by about 1.97% and 0.96% on the SEED and SEED-IV datasets, respectively. The accuracy decrease suggests that the graph convolutional feature extractor can capture the complex spatial relationships in the EEG data. Moreover, the extractor enhances the model’s performance on the emotion recognition task. When both the individual discriminator and the graph convolution feature extractor are removed, the model shows the worst performance, with an accuracy of only about 77.78% and 63.14% on the SEED and SEED-IV datasets. The results indicate that by combining the graph convolution feature extractor and the individual discriminator, the proposed method is able to take into account the differences in the individual emotion features. Moreover, it can model the complex relationships between different EEG channels in the process of emotion generation, so as to make the model achieve the best results.

### 4.5. Discussion and Analysis

**Figure 4:**
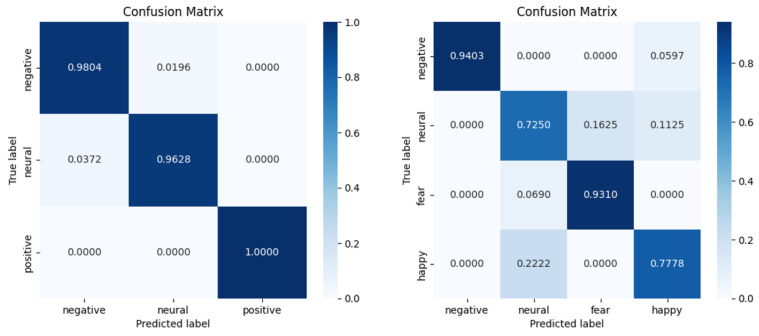
Confusion matrix of subject-dependent experiments on SEED and SEED-IV datasets.

**Figure 5:**
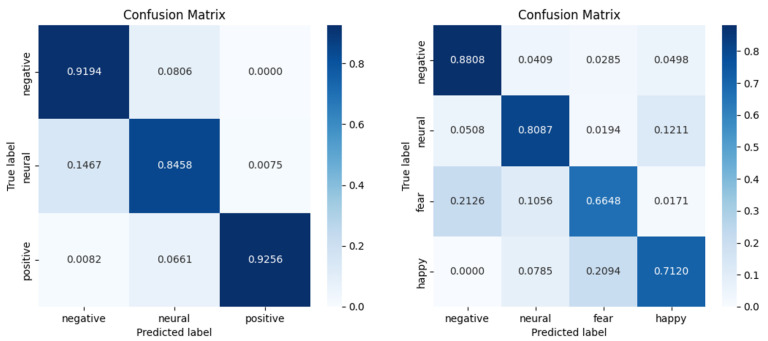
Confusion matrix of subject-independent experiments on SEED and SEED-IV datasets.

The confusion matrix of the proposed method is presented in Figure 4. The confusion matrix is the result for the subject-dependent experiments on the SEED and SEED-IV datasets. Moreover, Figure 5 shows the confusion matrix for the subject-independent experiments conducted on the two datasets. It can be seen from the two figures that for the SEED dataset, the model recognizes negative and positive emotions well in both experiments, and the recognition accuracy of neutral emotions is relatively poor. This could be due to the less distinctive characteristics of neutral emotions, or the similarity of their features to those of the other two emotions. It can be observed from the results on the SEED-IV dataset that the accuracy of recognizing negative emotions was greater than the other three emotions in both experimental scenarios. The result is similar to the findings in the SEED dataset. This indicates that negative emotions are more pronounced relative to the other emotions.

**Figure 6:**
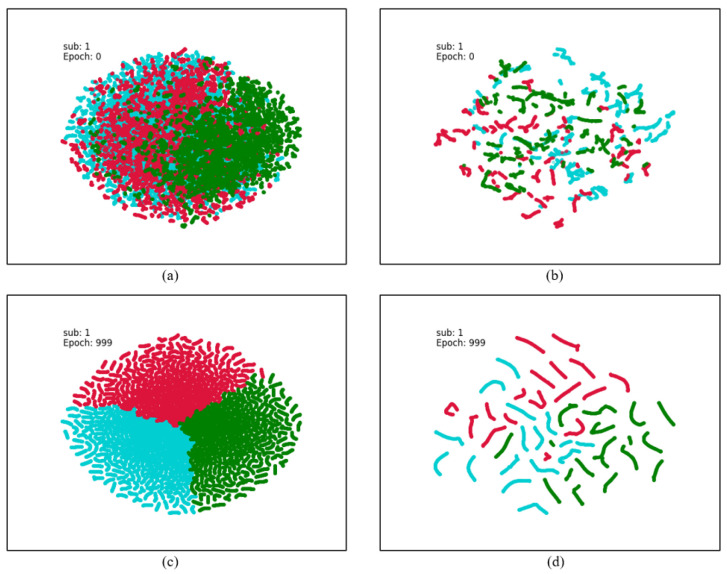
The t-SNE visualization of Subject 1 as the target domain in the subject-independent experiment on the SEED dataset. (**a**,**b**) are the source and target domain feature distributions obtained by the graph convolution feature extractor, and (**c**,**d**) are the source and target domain feature distributions after model training. Where blue color represents negative emotion, red color represents neutral emotion, and green color represents positive emotion.

To further analyze the effectiveness of TD-DANN, we employ t-distributed stochastic neighborhood embedding (t-SNE) to visualize the learned feature representations. As shown in Figure 6, the plots of (a) and (b) show the features extracted during subject-related and subject-independent experiments on the SEED dataset, and it can be found that there is an obvious overlap between different emotions, which suggests that the distribution of features on the original data is not consistent enough. In contrast, after TD-DANN training, the corresponding Figure 6c,d show a significant difference in the distribution of features across emotion categories in both experiments. This result suggests that the model can effectively categorize emotions. It is noteworthy that in the subject-independent experiment, i.e., Figure 6d, there is a significant difference in the distributions of the three emotion features, which indicates that the model has a good ability to generalize the emotion features of untrained subjects. However, there are still some emotional features mixed with each other on the boundary of neutral and negative emotions, a phenomenon that also corroborates with the results of the confusion matrix and suggests that there is room for improvement in the model’s recognition ability for neutral and negative emotions.

## 5. Conclusions

To acquire EEG signals for emotion recognition, non-invasive biosensors are utilized to capture electrical activity generated by neuronal processes in the brain. These non-invasive biosensors typically consist of electrodes placed on the scalp according to standardized positions, which detect minute voltage fluctuations resulting from synaptic activity. EEG emotion recognition decoding is widely recognized as a challenging task, primarily because brain signals are non-stationary, vary across individuals, and are often obscured by artifacts, making accurate emotion or intent interpretation difficult.

In this paper, we propose a two-discriminator domain adversarial neural network method aimed at addressing the problem of individual differences in EEG emotion recognition decoding tasks. The method extracts EEG signals through a graph convolution feature extractor, which effectively captures the spatial dependencies among the channels of EEG signals, thereby extracting more representative emotion features. The introduction of a domain discriminator and an individual discriminator helps to reduce the domain distribution gap, yielding generalized feature representations, while the individual discriminator learns unique individual features, enhancing the model’s adaptability to different individuals. Experiments are conducted on the SEED and SEED-IV datasets using both subject-dependent and subject-independent experimental settings. The results of the proposed method are compared with existing methods. The result analysis shows that our method shows promising performance. The proposed method provides a novel approach and solution to the problem of individual differences in EEG emotion recognition, particularly when dealing with cross-individual and cross-scenario emotion recognition tasks.

At the same time, the TD-DANN model has some limitations. For example, the distinction between the boundaries of neutral and negative emotions in subject-independent experiments is not clear enough, and the boundaries of the two emotions can be confused. In addition, the model training time is still difficult to meet the requirement of immediate detection of subjects’ emotions for practical applications. In the subsequent research, the shortening of the model training time on the basis of ensuring the classification accuracy can be explored to explore the application of instant emotion recognition.

## Figures and Tables

**Table 1:** Comparison of subject-dependent experimental accuracy (ACC) and standard deviation (STD) of models on SEED and SEED-IV datasets.

Method	ACC ± STD (%)	
	SEED	SEED-IV
SVM [10]	83.99±9.72	56.61±20.05
DBN [11]	86.08±8.34	66.77±7.38
DANN [26]	91.36±8.30	63.07±12.66
RGNN [29]	94.24±5.95	79.37±10.54
DGCNN [16]	90.40±8.49	69.88±16.29
Bi-DANN [18]	92.38±7.04	70.29±12.63
Bi-HDM [30]	93.12±6.06	74.35±14.09
PR-PL [31]	94.84±9.16	83.33±10.61
TD-DANN (Our model)	**98.45 ± 2.38**	**84.40 ± 8.70**

Bold text represents the best result.

**Table 2:** Comparison of subject-independent experimental accuracy (ACC) and standard deviation (STD) of models on SEED and SEED-IV datasets.

Method	ACC ± STD (%)	
	SEED	SEED-IV
SVM [10]	68.15±7.38	51.78±12.85
DANN [26]	83.81±8.56	58.87±8.13
RGNN [29]	85.30±6.72	73.84±8.02
DGCNN [16]	79.95±9.02	52.82±9.23
Bi-DANN [18]	83.28±9.60	65.59±10.39
Bi-HDM [30]	85.40±7.53	69.03±8.66
PR-PL [31]	85.56±4.78	74.92±7.92
TD-DANN (Our model)	**89.45 ± 5.87**	**77.13 ± 7.97**

Bold text represents the best result.

**Table 3:** Model ablation experiments on SEED and SEED-IV datasets.

Ablation	ACC ± STD (%)	
	SEED	SEED-IV
w/o Individual Discriminator	78.54±6.32	66.15±9.67
w/o Graph Convolutional Feature Extractor	87.48±5.79	76.17±7.28
w/o Graph Convolutional Feature Extractor and Individual Discriminator	77.78±7.20	63.14±8.40
TD-DANN	**89.45 ± 5.87**	**77.13 ± 7.97**

Bold text represents the best result.

## Data Availability

The SEED dataset analyzed in this study is available from https://bcmi.sjtu.edu.cn/home/seed/seed.html (accessed on 8 May 2025), and the SEED-IV dataset is available from https://bcmi.sjtu.edu.cn/home/seed/seed-iv.html (accessed on 11 May 2025).

## Abbreviations

The following abbreviations are used in this manuscript:

ER	Emotion recognition
MEG	Magnetoencephalograph
SVM	Support vector machine
DBN	Deep belief network
PSD	Power spectral densit
CNN	Convolutional neural network
TD-DANN	Two-discriminator domain adversarial neural network
GCN	Graph convolutional network
DANN	Domain adversarial neural network
